# Macrophage migration inhibitory factor mediates protease‐activated receptor 4‐induced bladder pain through urothelial high mobility group box 1

**DOI:** 10.14814/phy2.13549

**Published:** 2017-12-21

**Authors:** Fei Ma, Dimitrios E. Kouzoukas, Katherine L. Meyer‐Siegler, David E. Hunt, Lin Leng, Richard Bucala, Pedro L. Vera

**Affiliations:** ^1^ Research and Development Lexington Veterans Affairs Medical Center Lexington Kentucky; ^2^ Department of Physiology University of Kentucky Lexington Kentucky; ^3^ Saha Cardiovascular Research Center University of Kentucky Lexington Kentucky; ^4^ Department of Natural Sciences St. Petersburg College St. Petersburg Florida; ^5^ Department of Internal Medicine Yale University New Haven Connecticut; ^6^ Department of Surgery University of Kentucky Lexington Kentucky; ^7^ Present address: Department of Molecular Pharmacology and Therapeutics Loyola University Chicago Maywood Illinois

**Keywords:** Abdominal mechanical hypersensitivity, bladder pain, HMGB1, MIF, PAR4

## Abstract

Macrophage migration inhibitory factor (MIF) mediates pain although the mechanisms are not well understood. Urothelial activation of protease activated receptor 4 (PAR4) results in urothelial MIF release, urothelial high mobility group box 1 (HMGB1) release and bladder pain in mice without bladder inflammation. All three effects are prevented by MIF inhibition while intravesical disulfide HMGB1 alone can induce bladder pain. This study utilizes genetic MIF deletion to determine whether MIF mediates PAR4‐induced bladder pain and is upstream of HMGB1‐induced bladder pain. Wild type (C57/BL6) and MIF knockout (KO) mice were treated with intravesical PAR4 activating peptide or disulfide HMGB1 and tested for abdominal mechanical hypersensitivity at baseline (before treatment) and 24 h after injection. Micturition parameters and bladder histology were examined after behavioral test. Real‐time PCR and western blotting measured HMGB1 mRNA and protein levels in the bladders of naïve wild type and MIF KO mice, while immunofluorescence measured HMGB1 protein levels in the urothelium of both strains. Intravesical PAR4 activation resulted in abdominal mechanical hypersensitivity in wild‐type mice but not MIF KO mice. Intravesical disulfide HMGB1 induced abdominal mechanical hypersensitivity in both strains. Neither treatment resulted in significant changes in micturition or bladder histology in either strain. HMGB1 mRNA and protein levels were higher in MIF KO mouse bladders and the urothelium of MIF KO bladder had greater immunostaining than the wild‐type strain. MIF is a pivotal molecule mediating PAR4‐induced bladder pain and regulating urothelial HMGB1 production and release to elicit bladder pain.

## Introduction

Macrophage migration inhibitory factor (MIF) is a cytokine that is pre‐formed in various types of cells and stored in vesicles to be released upon appropriate stimuli (Lolis and Bucala 2003). MIF is known as a key player in innate and adaptive immune responses (Flaster et al. 2007). Recent evidence indicates that MIF is involved in nociception, either by mediating pain induced by inflammation or nerve injury (Morand et al. 2002; Aloisi et al. 2005; Ellis et al. 2014) or by eliciting pain directly (Alexander et al. 2012; Lerch et al. 2014).

MIF is expressed in central nervous system cells (including neurons and glial cells) and also expressed in peripheral tissues such as macrophages, choroid plexus, anterior pituitary, gastric, colonic, and urinary epithelium (Meyer‐Siegler 2001; Lue et al. 2002; Vera and Meyer‐Siegler 2003). MIF release was observed from bladder urothelium and peripheral as well as central nervous system in a different inflammatory model of cystitis in rodents (Vera and Meyer‐Siegler 2003; Meyer‐Siegler and Vera 2004; Vera et al. 2010).

Animal models of painful bladder syndrome/interstitial cystitis (PBS/IC) usually employ chemical‐initiated bladder injury or inflammation (Saban et al. 2001; Westropp and Buffington 2002; Cruz et al. 2005). We recently reported that activation of urothelial protease activated receptor 4 (PAR4) receptors results in urothelial MIF release, urothelial high mobility group box 1 (HMGB1) release and abdominal mechanical hypersensitivity (indirect index of bladder pain) in mice without resulting in other inflammatory changes or altering voiding behavior. These three effects were blocked by a MIF antagonist suggesting that MIF mediated HMGB1 release and abdominal hypersensitivity (Kouzoukas et al. 2015).

HMGB1, a ubiquitous and abundant non‐histone nuclear chromatin‐binding protein, is actively secreted in response to inflammatory signals and is recognized as mediating pain (Agalave and Svensson 2015). In fact, intravesical administration of HMGB1 (in its disulfide form, dsHMGB1) induced abdominal mechanical hypersensitivity through urothelial Toll‐like receptor 4 (TLR4) without causing bladder inflammation or micturition changes (Ma et al. 2017).

We hypothesized that MIF is a pivotal molecule in PAR4‐activation induced bladder pain and that MIF mediates urothelial HMGB1 release to bind to urothelial HMGB1 receptors resulting in bladder pain. However, it is not clear if MIF deletion will break the signaling pathway in this PAR4 activation model of bladder pain. This study utilized global MIF knockout (KO) to test this hypothesis and investigate more precisely the role of MIF in PAR4‐induced bladder pain and urothelial HMGB1 regulation.

## Material and Methods

### Animals

All animal experiments were approved by the Lexington Veterans Affairs Medical Center Institutional Animal Care and Use Committee (VER‐11‐016‐HAF) and performed according to the guidelines of the National Institutes of Health. MIF knockout mice were obtained from Yale University and bred in our animal facility (Fingerle‐Rowson et al., 2003) and wild‐type C57/BL6 mice were purchased from Jackson Laboratory (Jackson Laboratory, Bar Harbor, ME).

### Abdominal mechanical hypersensitivity test

Abdominal mechanical hypersensitivity was tested in mice as previously described (Kouzoukas et al. 2015). Briefly, von Frey filaments of ascending bending force (0.008, 0.02 0.04, 0.07 g) were pressed to the lower abdominal region in trials of 10 before (baseline) and 24 h after intravesical instillation of either PAR4 scramble (H‐Tyr‐Ala‐Pro‐Gly‐Lys‐Phe‐NH_2_, PAR‐3933‐PI) or PAR4‐activating peptide (PAR4‐AP, a short synthetic peptide that stimulates PAR4 in a protease‐independent manner, H‐Ala‐Tyr‐Pro‐Gly‐Lys‐Phe‐NH_2_, PAR‐3674‐PI, Peptides International, Louisville, KY) (100 *μ*mol/L/150 *μ*L PBS) or dsHMGB1 (10 *μ*g/150 *μ*L PBS, HM‐121, HMGBiontech, Italy) to detect referred bladder pain. Positive response was defined as any one of three behaviors: (1) licking the abdomen, (2) flinching/jumping, or (3) abdomen withdrawal. Mice responding more than 30% to the weakest filament (0.008 g) during baseline testing were excluded from the study.

### Voided stain on paper (VSOP)

Micturition volume and frequency were measured in awake mice as previously described (Sugino et al. 2008; Ma et al. 2017). Briefly, mice were gavaged with water (50 *μ*l/g body weight) to induce diuresis, and then placed in a plastic enclosure. Mice were free to move and filter paper was placed under the animal to collect urine during a 2 h observation period. Micturition volumes were determined by linear regression using a set of known volumes. Micturition frequency was defined as total number of micturitions in two hours.

### Histology and immunohistochemistry

Bladder paraffin sections (5 *μ*m) were processed for routine hematoxylin and eosin (H&E) staining. H&E stained sections were evaluated by a pathologist blinded to the experimental treatment and scored for edema and inflammation according to the following scale: 0, No edema and no infiltrating cells; 1, Mild submucosal edema and no inflammatory cells; 2, Moderate edema and several inflammatory cells; 3, Frank edema, vascular congestion and many inflammatory cells.

For immunohistochemistry, batch‐stained paraffin sections (*N* = 6/group) were blocked (5% goat serum, 0.2% Triton X‐100 in PBS, 30 min at room temp.), then incubated overnight at 4°C with rabbit polyclonal anti‐HMGB1 antibody (1:100; ab18256; Abcam, Cambridge, MA). Immunoreactivity was detected with goat anti‐rabbit TRITC‐labeled secondary antibody (1:100 in PBS with 1% goat serum, 0.2% Triton X‐100; 1 h at room temp.; Jackson ImmunoResearch, Inc., West Grove, PA) before cover‐slipping (Vectashield, Vector Laboratories, Burlingame, CA). Computer‐assisted densitometry of HMGB1 immunostaining intensity was performed on images captured using a LEICA DMI4000B microscope equipped with the LAS V4 program and ImageJ (NIH, Bethesda, MD).

### Western blotting

Proteins extracted from bladder tissue of naïve wild type and MIF knockout mice were separated using a 4–15% Mini‐PROTEAN TGX precast polyacrylamide gel (Bio‐Rad, Hercules, CA). After electrophoresis, separated proteins were transferred to a polyvinylidene difluoride membrane. HMGB1 protein bands were visualized using a rabbit polyclonal primary antibody (ab18256; Abcam, Cambridge, MA; 1:4000), along with GAPDH as an internal control (A00915, GenScript, Piscataway, NJ; 1:2500), a biotinylated anti‐rabbit secondary antibody (Vector Labs, Burlingame, CA; 1:400), streptavidin‐HRP conjugates and chemiluminescent substrate (Pierce, Rockford, IL). Band densitometry was performed using ImageJ (NIH, Bethesda, MD).

### Real‐time PCR

Total RNA was extracted from wild type and MIF knockout mouse bladder tissue through Trizol (15596026, ThermoFisher Scientific, Grand Island, NY), DNA removed by DNase, and reversed transcribed to cDNA (A3500, Promega, Madison, WI). SYBR green (4472903, ThermoFisher Scientific, Grand Island, NY) was utilized with primers (HMGB1, PPM05059F; Rn18s, PPM72041A, Qiagen, Germantown, MD) to quantified level of mRNA in bladder tissue from wild type and MIF knockout mice. 18S rRNA was used as the internal control.

### Statistical analyses

Changes in positive response frequency (%) to von Frey stimulation at baseline and 24 h after treatment were evaluated using a within subject two‐way (Time x Filament Strength) ANOVA. When the Time factor (pre vs. post) was significant, differences at each filament strength were compared (pre vs. post) using t‐tests with a multiple comparison adjustment (Holm‐Sidak). Micturition parameters (volume; frequency) were analyzed using a two‐way ANOVA (treatment vs. strain).

## Results

### Lack of response to intravesical PAR4‐AP in MIF KO mice

**Figure 1:**
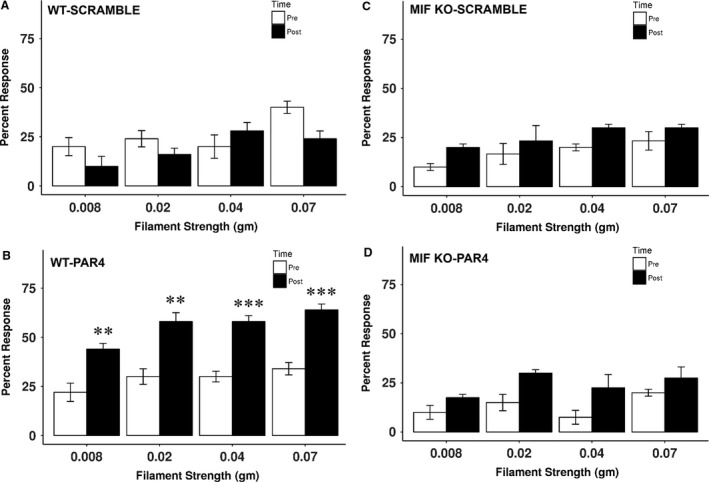
Abdominal mechanical sensitivity after PAR4 in WT and MIF KO mice. PAR4 activating peptide or PAR4 scramble was intravesically injected into WT and MIF knockout mice. Abdominal mechanical sensitivity was measured 24 h after instillation. (A) PAR4 scramble did not induce mechanical response change in WT mice (*n* = 6). (B) PAR4 significantly increased number of responses to abdominal mechanical stimulation in WT mice (*n* = 5). Neither PAR4 scramble (*n* = 3) (C) nor PAR4 (*n* = 4) (D) caused abdominal mechanical hypersensitivity in MIF knockout mice. **P <* *0.01; ***P <* *0.001.

PAR4‐AP was administered intravesically to wild type and MIF knockout mice after baseline von Frey measurement. Twenty‐four hours after injection, abdominal mechanical sensitivity to von Frey filaments was tested again. In wild‐type mice, PAR4‐AP induced increased number of responses to von Frey filaments on abdominal/perianal area. Wild‐type mice showed significant increases in percentage of responses to all strength of filaments (0.008, 0.02, 0.04, and 0.07 g) compared to baseline (*P *<* *0.01 or 0.001 on all comparison between before and after PAR4‐AP injection of all four filaments, *n* = 5, Fig. 1B). PAR4 scramble injection did not develop any hypersensitivity on abdomen in wild‐type mice (*n* = 6, Fig. 1A). However, PAR4‐AP did not cause any abdominal mechanical hypersensitivity in MIF knockout mice. For each filament, there was no increase in percent response to abdominal mechanical stimuli at 24 h after PAR4‐AP injection compared to baseline (*P *>* *0.05 on each filament along each time point, *n* = 4, Fig. 1D). PAR4 scramble as control also did not cause any behavioral changes in MIF knockout mice (*n* = 3, Fig. 1C).

### Intravesical dsHMGB1 induced abdominal mechanical hypersensitivity in MIF KO mice

**Figure 2:**
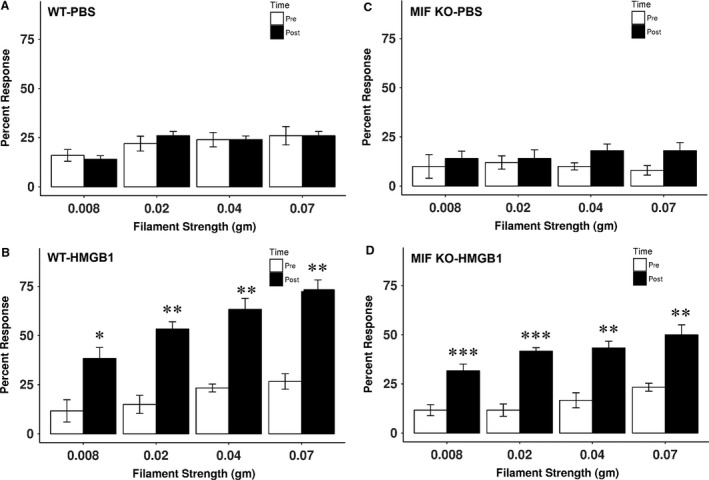
Abdominal mechanical hypersensitivity after dsHMGB1 in WT and MIF KO mice. DsHMGB1 or vehicle (PBS) was intravesically injected into WT and MIF knockout mice. Abdominal mechanical sensitivity was measured 24 h after instillation. (A) PBS did not induce mechanical response change in WT mice (*n* = 5). (B) DsHMGB1 significantly increased number of responses to abdominal mechanical stimulation in WT mice (*n* = 6). (C) PBS did not cause mechanical stimulation response change in MIF knockout mice (*n* = 5). (D) DsHMGB1 significantly increased number of responses to abdominal mechanical stimulation in MIF knockout mice (*n* = 6). *P <* *0.05; **P <* *0.01; ***P <* *0.001.

DsHMGB1 was administered intravesically to wild type and MIF knockout mice after baseline measurement. Twenty‐four hours after injection, abdominal mechanical sensitivity to von Frey filaments was tested again. In wild‐type mice, dsHMGB1 induced increased number of responses to von Frey filaments on abdominal/perianal area. Wild‐type mice showed significant increases in percentage of responses to all strength of filaments (0.008, 0.02, 0.04, and 0.07 g) compared to baseline (*P *<* *0.05 or 0.01 on all comparison between before and after dsHMGB1 injection of all four filaments, *n* = 6, Fig. 2B). Vehicle (PBS) injection did not develop any abdominal hypersensitivity in wild‐type mice (*n* = 5, Fig. 2A). Similarly, dsHMGB1 caused abdominal mechanical hypersensitivity in MIF knockout mice to von Frey filaments on abdominal/perianal area by increasing percentage of responses to all strength of filaments (0.008, 0.02, 0.04, and 0.07 g) compared to baseline (*P *<* *0.01 or 0.001 on all comparison between before and after dsHMGB1 injection of all four filaments, *n* = 6, Fig. 2D). There was no increase in percent response to abdominal mechanical stimuli at 24 h after vehicle injection compared to baseline (*P *>* *0.05 on each filament along each time point) (*n* = 5, Fig. 2C).

### Micturition changes after intravesical PAR4‐AP or dsHMGB1 injection

Micturition parameters were recorded 24 h after PAR4‐AP or dsHMGB1 injection. PAR4‐AP did not result in statistically significant changes in urine volume (in *μ*L) or frequency when compared to scrambled peptide (control) in either of the mouse strains (Table 1A). Moreover, intravesical dsHMGB1 treatment produced no statistically significant changes on micturition volume or frequency when compared to vehicle treatment in either strain (Table 1B). Micturition volume was significantly larger in MIF knockout mice receiving control treatment (632 ± 101 for PAR4 scramble and 675 ± 66.9 for PBS; *P *<* *0.01; Table 1) compared to wild‐type mice receiving control treatments (257 ± 11.4 for PAR4 scramble and 301 ± 35.2 for PBS; Table 1). Similarly, micturition frequency was lower in MIF knockout mice (1.0 ± 0.0 for PAR4 scramble and 1.2 ± 0.2 for PBS; *P *<* *0.01; Table 1) compared to wild‐type mice (3.3 ± 0.3 for PAR4 scramble and 3.0 ± 0.3 for PBS; Table 1).

**Table 1:** Micturition after intravesical administration in WT and MIF KO mice

	WT		MIF KO	
	Volume (μL)	Frequency	Volume (μL)	Frequency
**(A)**				
PAR4 scramble	257 ± 11.4	3.3 ± 0.3 (*n* = 6)	**632 ± 101** **	**1.0 ± 0.0** ** (*n* = 3)
PAR4	235 ± 24.3	4.6 ± 0.9 (*n* = 5)	417 ± 84.9	1.8 ± 0.5 (*n* = 4)
**(B)**				
PBS	301 ± 35.2	3.0 ± 0.3 (*n* = 5)	**675 ± 66.9** **	**1.2 ± 0.2** ** (*n* = 5)
dsHMGB1	265 ± 25.5	4.0 ± 1.0 (*n* = 6)	485 ± 97.3	1.8 ± 0.4 (*n* = 6)

(A) **P < 0.01 compared to WT PAR4 scramble (B) **P <* *0.01 compared to WT PBS; value: mean ± SE. Bold indicates significance value.

### Histology after intravesical PAR4‐AP or dsHMGB1 injection

H&E stained bladder sections from wild‐type and MIF knockout mice that received intravesical PAR4‐AP or dsHMGB1 were examined by a pathologist blinded to the treatment and scored for inflammation and edema changes. Neither PAR4‐AP (Figure S1) nor dsHMGB1 (Figure S2) treatment produce statistically significant changes in inflammation or edema in either of the strains (Table 2).

**Table 2:** Histology after intravesical administration in WT and MIF KO mice

	WT		MIF KO	
	Inflammation	Edema	Inflammation	Edema
(A)				
PAR4 scramble	0.0 ± 0.0	0.0 ± 0.0 (*n* = 6)	0.7 ± 0.3	0.3 ± 0.3 (*n* = 3)
PAR4	0.0 ± 0.0	0.0 ± 0.0 (*n* = 5)	0.8 ± 0.5	0.8 ± 0.5 (*n* = 4)
(B)				
PBS	1.0 ± 0.6	1.0 ± 0.6 (*n* = 5)	0.8 ± 0.2	1.2 ± 0.4 (*n* = 5)
dsHMGB1	0.5 ± 0.5	1.0 ± 0.5 (*n* = 6)	0.7 ± 0.3	1.3 ± 0.4 (*n* = 6)

value: mean ± SE.

### Increases in HMGB1 mRNA and protein levels in the bladder of MIF KO mice

**Figure 3:**
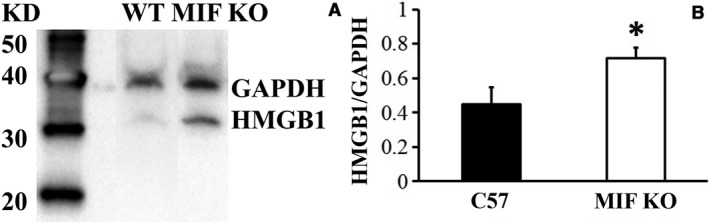
Elevated HMGB1 in MIF KO by western blot. HMGB1 protein was measured from bladder extraction. (A) HMGB1 band was more intensive in MIF knockout mice than that in WT mice. (B) Histogram showed that MIF knockout bladder HMGB1 protein level is significantly higher than that in WT mice when normalized to GAPDH. *P <* *0.05.

**Figure 4:**
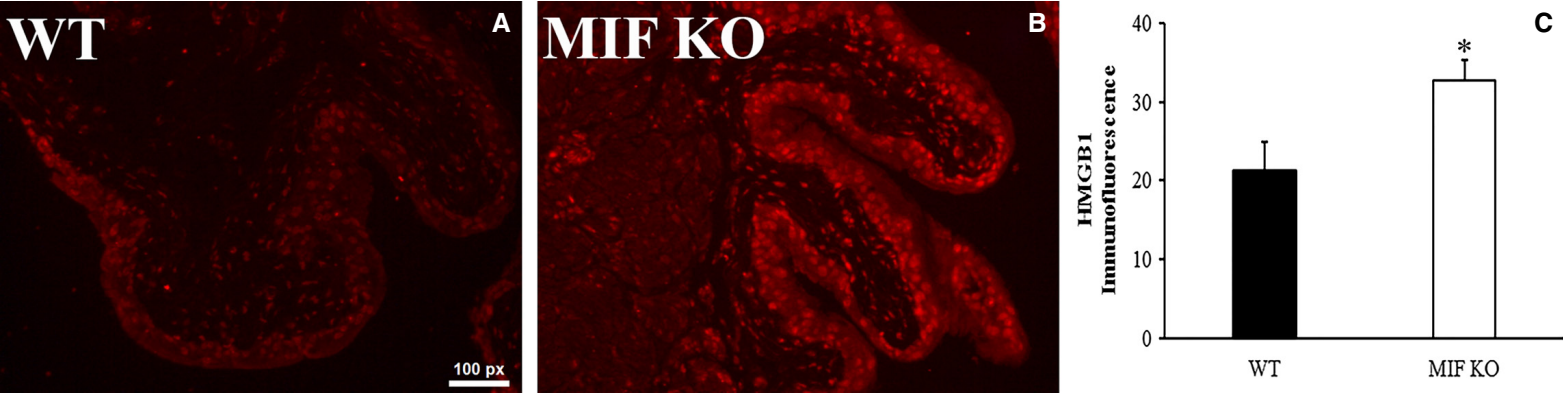
Higher HMGB1 in MIF KO bladder urothelium by immunofluorescence. Urothelial HMGB1 expression was measured by immunostaining. (A) HMGB1 was expressed in bladder urothelium in WT mice. (B) HMGB1 expression was higher in MIF knockout mice shown by brighter staining. (C) Histogram showed that MIF knockout mice have more HMGB1 expression in bladder urothelium than that in WT mice. *P <* *0.05.

Bladder levels of HMGB1 mRNA and protein were examined in both wild type and MIF knockout mice. Real‐time PCR result showed that basal level of HMGB1 mRNA in MIF knockout mice is 16.4 times of that in wild‐type mice when normalized to 18S rRNA (*P < *0.05). Protein levels of bladder HMGB1 were tested by both western blot and immunofluorescence. Western blots showed higher levels of HMGB1 (normalized to GAPDH) intensity in bladders from MIF knockout mice than from wild‐type mice (*P < *0.05;) (Fig. 3A, B). HMGB1 immunofluorescence (Fig. 4) showed stronger fluorescence in bladder urothelium in MIF knockout mice (Fig. 4B) than that in wild‐type mice (Fig. 4A). Densitometry showed that this difference was statistically significant (*P < *0.05) (Fig. 4C).

## Discussion

The present results clearly demonstrate that MIF is a signaling molecule mediating bladder pain. Intravesical PAR4 activation resulted in abdominal mechanical hypersensitivity in wild‐type mice, in agreement with our earlier reports (Kouzoukas et al. 2015, 2016), but not in MIF knockout mice. Moreover, dsHMGB1 induced abdominal mechanical hypersensitivity in both wild‐type and MIF knockout mice indicating that HMGB1 is downstream of MIF.

Histological scoring showed no significant changes in the bladder 24 h after either PAR4 or dsHMGB1 instillation, as we reported earlier (Kouzoukas et al. 2015, 2016; Ma et al. 2017). H&E staining showed no infiltrating cells with minimal to mild bladder edema and stromal reactive changes in some mice (reactive submucosal fibrosis with lamina propria expansion).

In this study, we again observed no significant differences in awake micturition volume or frequency as a result of intravesical treatment in either of the strains and this agrees with our previous reports of no micturition changes after intravesical either PAR4‐AP or dsHMGB1 (Kouzoukas et al. 2015, 2016; Ma et al. 2017). There were significant strain differences in the control groups (intravesical PBS or PAR4 scramble) with MIF knockout mice showing significant increased bladder capacity (almost twofold). These changes are unlikely to be due to PBS or scrambled PAR4 peptide (since PBS or PAR4 scramble has no effect on micturition in naïve control MIF knockout mice (data not shown)). But do suggest that MIF plays a role in bladder sensation. This last possibility warrants further study.

We also examined if there were any basal differences in bladder HMGB1 mRNA or protein levels between wild‐type and MIF knockout mice. Our analyses showed increased HMGB1 mRNA and protein levels in the bladder of MIF knockout mice compared to wild‐type mice. Furthermore, the urothelium had higher HMGB1 protein levels (detected by immunohistochemistry) in MIF knockout than in wild‐type mice. It is uncertain whether urothelial HMGB1 protein increase is due to more production or less release in MIF knockout mice and is likely a reflection of both effects. Nevertheless, the finding that MIF controls HMGB1 mRNA expression, protein levels and protein release in the bladder is a novel finding.

We previously determined that MIF is a likely factor mediating PAR4‐induced mechanical hypersensitivity since PAR4 evoked urothelial MIF release and a MIF inhibitor (ISO‐1) blocked PAR4‐induced abdominal mechanical hypersensitivity (Kouzoukas et al. 2015). We also showed that PAR4 activation leads to urothelial MIF release that likely acts on MIF receptors to induce urothelial HMGB1 release (Kouzoukas et al. 2016). MIF‐induced HMGB1 release is in agreement with a recent in vitro study showed that MIF promotes HMGB1 release in breast cancer cells (Lv et al. 2016). We also demonstrated that intravesical dsHMGB1 (but not the thiol form) elicited mechanical hypersensitivity through activation of toll‐like receptor 4 (TLR4) receptors (Ma et al. 2017).

**Figure 5:**
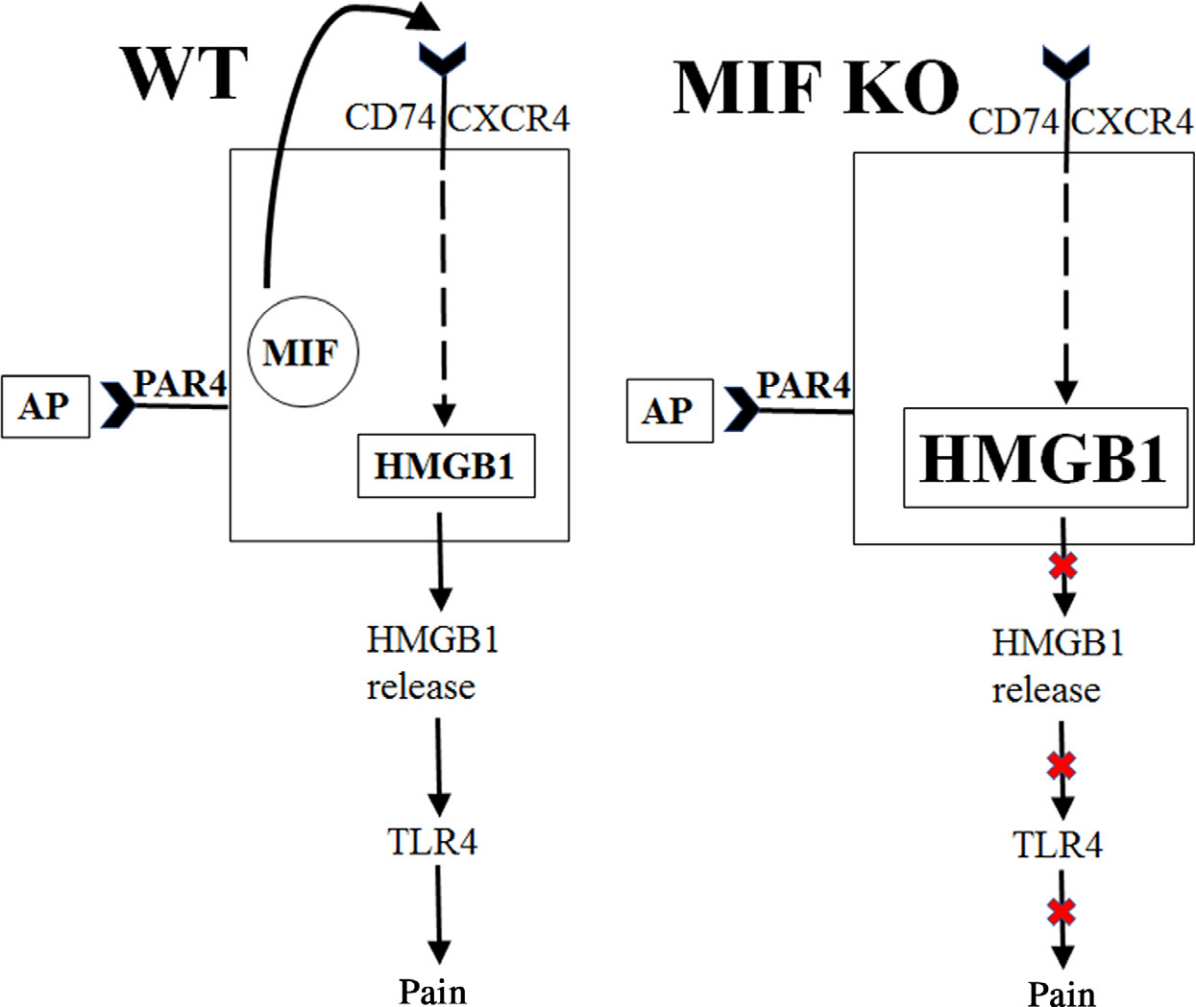
Diagram showing MIF mediates PAR4‐induced bladder pain through HMGB1 release in wild type and MIF knockout mice.

Combining our previous findings with current results we hypothesize that MIF directly regulates HMGB1 expression and release in the bladder to mediate bladder pain (Fig. 5). Current results demonstrate that MIF is upstream of HMGB1 and removing MIF (as in the case of MIF knockout) prevents PAR4‐induced abdominal mechanical sensitivity. We previously showed reduced protein levels of MIF and HMGB1 in the urothelium after PAR4 activation with concomitant MIF and HMGB1 release from bladder (Kouzoukas et al. 2015, 2016). We propose that in MIF knockout mice HMGB1 is not released from the urothelium after PAR4‐AP. Reduced HMGB1 release from the urothelium coupled with increased HMGB1expression in MIF knockout mice may thus lead to protein accumulation in the urothelium.

Our current findings present evidence that MIF has a pivotal role in visceral pain mediation in addition to its involvement in somatic pain (Wang et al. 2010; Alexander et al. 2012). So, we report that MIF is a major molecule mediating HMGB1 release in bladder urothelium in the model of PAR4‐induced bladder pain that does not have other signs of bladder inflammation. MIF and/or HMGB1 are potential novel targets in the treatment of bladder pain independent of inflammation and may represent new approaches to understanding and treatment of painful bladder syndrome/interstitial cystitis.

## Conflicts of Interest

The authors declare that there are no conflicts of interest.

## Data Accessibility

## Supporting information




**Figure S1.** Bladder histology after PAR4 in WT and MIF KO mice**.** PAR4 or PAR4 scramble was intravesically injected into WT and MIF knockout mice.

**Figure S2.** Bladder histology after dsHMGB1 in WT and MIF KO mice. DsHMGB1 or vehicle (PBS) was intravesically injected into WT and MIF knockout mice.
