# Supplementary figures and images for: Macrophage migration inhibitory factor mediates protease‐activated receptor 4‐induced bladder pain through urothelial high mobility group box 1
Source: Physiol Rep. 2017 Dec 21;5(24):e13549. doi: 10.14814/phy2.13549 (PMC5742707; doi:10.14814/phy2.13549)

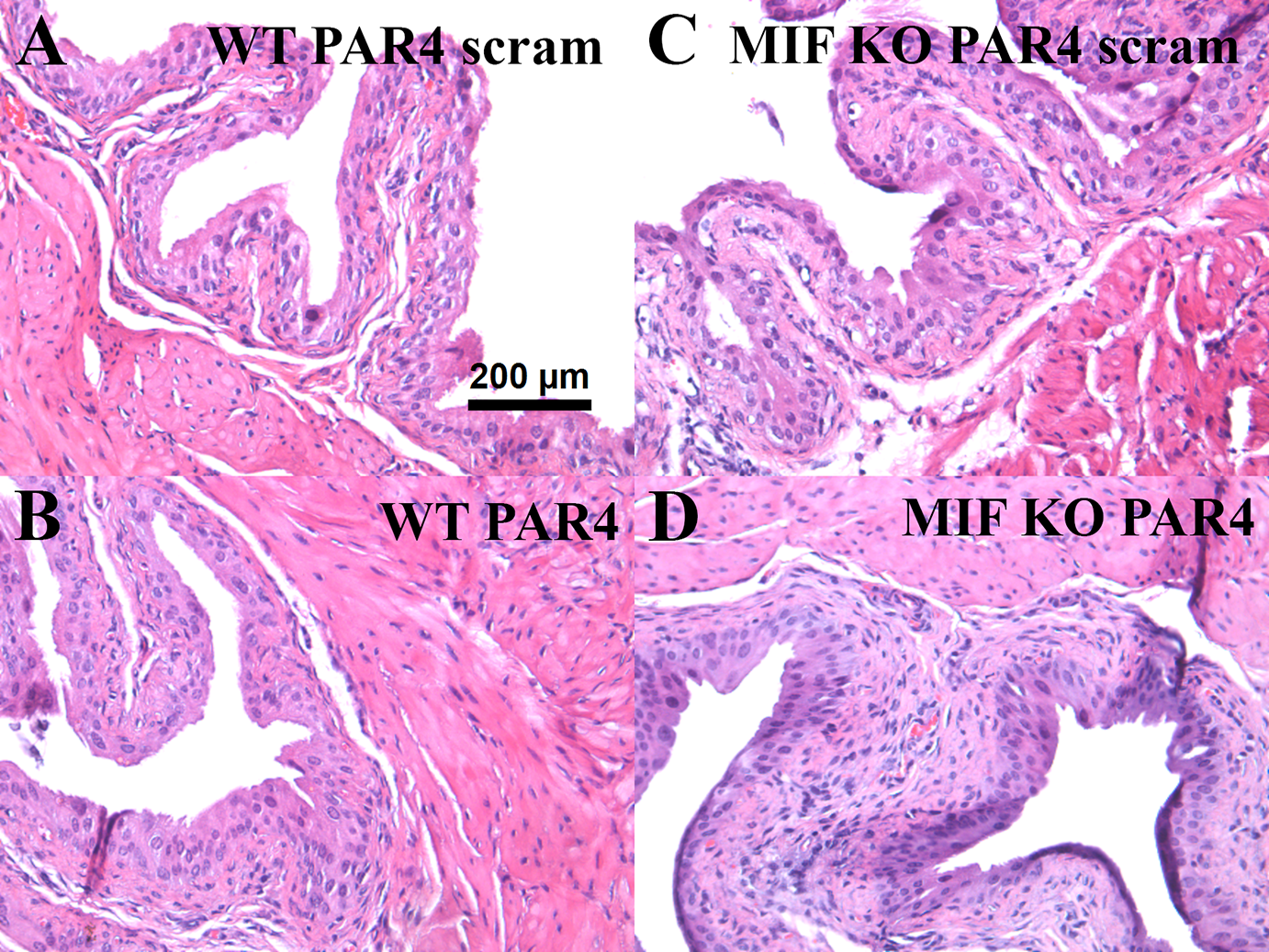

Supplement: Supplementary file 1 — Figure S1. Bladder histology after PAR4 in WT and MIF KO mice. PAR4 or PAR4 scramble was intravesically injected into WT and MIF knockout mice. [file PHY2-5-e13549-s001.tif]

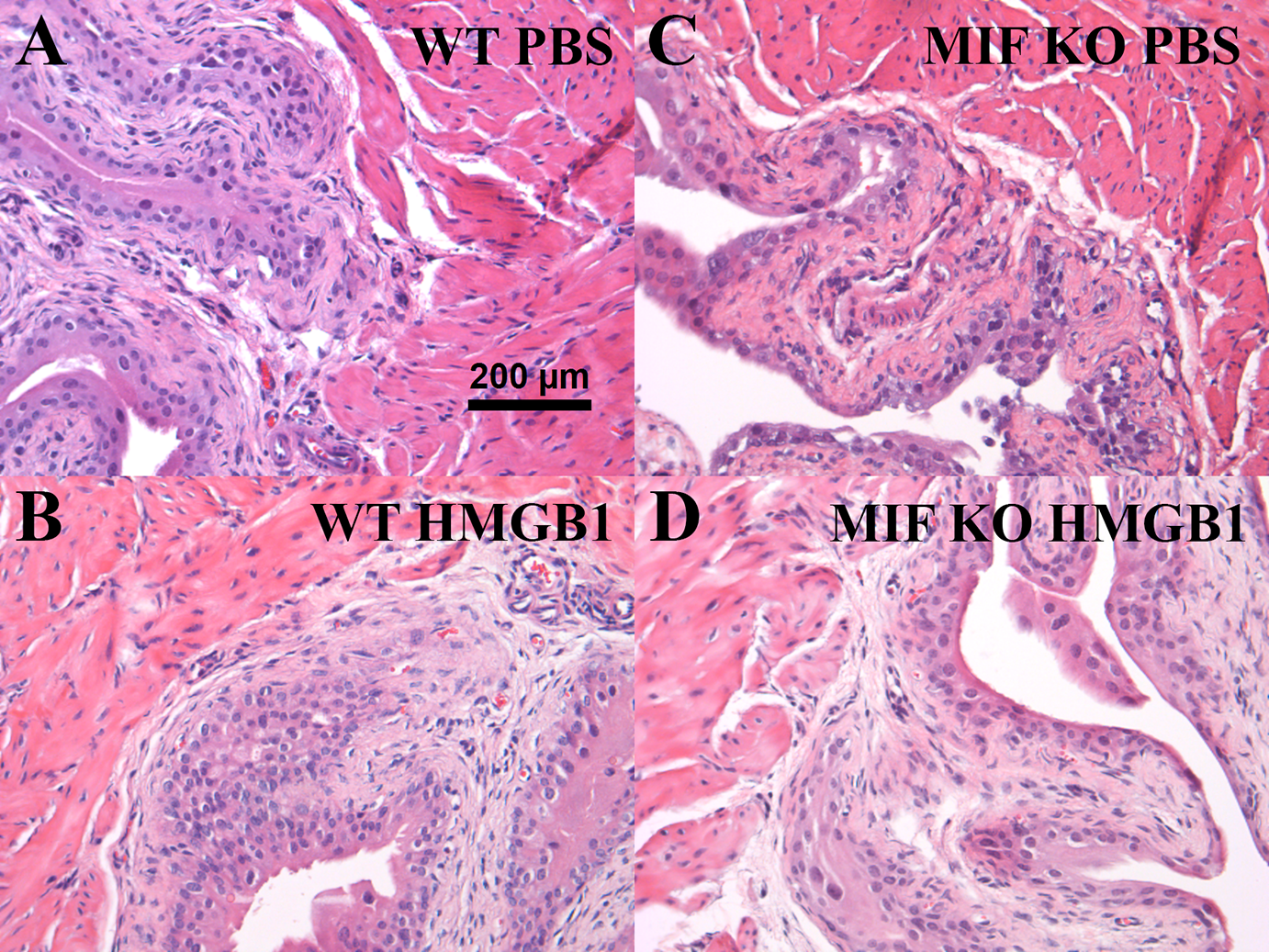

Supplement: Supplementary file 2 — Figure S2. Bladder histology after dsHMGB1 in WT and MIF KO mice. DsHMGB1 or vehicle (PBS) was intravesically injected into WT and MIF knockout mice. [file PHY2-5-e13549-s002.tif]
