# Supplementary material for: Macrophage migration inhibitory factor mediates protease‐activated receptor 4‐induced bladder pain through urothelial high mobility group box 1
Source: Physiol Rep. 2017 Dec 21;5(24):e13549. doi: 10.14814/phy2.13549 (PMC5742707; doi:10.14814/phy2.13549)
Supplement: Supplementary file 3 [file PHY2-5-e13549-s003.docx]

**Supplemental figure 1 Bladder histology after PAR4 in WT and MIF KO mice** PAR4 or PAR4 scramble was intravesically injected into WT and MIF knockout mice. Bladder histology was examined 24 hours after instillation. (A) PAR4 scramble did not induce histological change in WT mice. (B) No histological change was found after PAR4 instillation in WT mice. (C) PAR4 scramble did not induce histological change in MIF knockout mice. (D) No histological change was found after PAR4 instillation in MIF knockout mice.

**Supplemental figure 2 Bladder histology after dsHMGB1 in WT and MIF KO mice** DsHMGB1 or vehicle (PBS) was intravesically injected into WT and MIF knockout mice. Bladder histology was examined 24 hours after instillation. (A) PBS did not induce histological change in WT mice. (B) No histological change was found after dsHMGB1 instillation in WT mice. (C) PBS did not induce histological change in MIF knockout mice. (D) No histological change was found after dsHMGB1 instillation in MIF knockout mice.
